# Confrontations and wear and tear of academic life: a proposal for an undergraduate course^*^

**DOI:** 10.1590/1980-220X-REEUSP-2025-0115en

**Published:** 2026-01-16

**Authors:** Ricardo Santoro, Carla Andrea Trapé, Célia Maria Sivalli Campos, Ana Claudia Camargo Gonçalves Germani, Iasmin Cordeiro de Oliveira, Paulo de Melo Guimarães

**Affiliations:** 1 Universidade de São Paulo, Escola de Enfermagem, Departamento de Enfermagem em Saúde Coletiva, São Paulo, SP, Brazil.; 2 Universidade de São Paulo, Faculdade de Medicina, Departamento de Medicina Preventiva, São Paulo, SP, Brazil.

**Keywords:** Universities, Stress, Psychological, Policy, Community-Based Participatory Research

## Abstract

**Objective:**

To identify the contributions of the course “Coping with the wear and tear of university life” to promote the joint development of strategies by students to strengthen their academic experience.

**Method:**

This is a descriptive study with a qualitative approach based on participant research, the product of a master’s thesis. Students were asked to answer open-ended questions related to the causes of the stresses of academic life and coping strategies applied on the first and last days of class.

**Results and Discussion:**

Nine undergraduate students participated in the course. At the end of the course, the students’ conceptions of the wear and tear of academic life and its causes began to include the structural dimension of the reproduction of neoliberal values in the university, and coping strategies focused on those related to student movements and collective participation.

**Conclusion:**

The course contributed to the understanding of the roots of the wear and tear of the students’ academic experience and the development of proposals for collective coping.

## INTRODUCTION

The university experience has been characterized by an overload of production, in a context marked by the need to respond to various demands, which generate competitiveness and insecurity about the future, producing wear and tear manifested as psychological distress^(1)^.

A survey of 136,000 undergraduate students found that 80% of these students reported anxiety, feelings of discouragement and lack of motivation, insomnia, helplessness in the family environment, loneliness, eating disorders, panic syndrome, and suicidal thoughts^(2)^. A study conducted with undergraduate students in the health field found worrying results; 70.7% showed signs of psychological distress, with a 2.4 times greater chance of occurring in students from families in social groups with more unstable working and living conditions^(3)^.

This study is based on the theoretical and methodological foundations of the field of Collective Health, which defines the results of the health-disease process as the result of the clash between the potential for strengthening and deterioration arising from the social reproduction profiles characteristic of different social classes^(4)^.

It is assumed that wear and tear is experienced unequally by students from different social conditions^(5)^ and, considering that most undergraduates are young, the different social reproduction profiles determine the different profiles of university youth, with heterogeneous working, living, and health conditions^(5)^. Thus, understanding youth from the perspective of social reproduction, identifying potential for strengthening and wear and tear, makes it possible to overcome hegemonic theories of the health-disease process^(6)^ that disregard the relationship between class insertion and the health conditions of the population.

From this perspective, it is important to consider that young people from different social classes experience the social context unequally and, consequently, the university context^(6)^. Suffering is the expression in the body of what is produced in neoliberal capitalist society and reproduced in universities, affecting undergraduates and intensifying the wear and tear generated by pressures resulting from high levels of competitiveness, hopelessness in entering the job market, demands for increased academic productivity, among others^(7)^.

Capitalist sociability reinforces values associated with individual success, wealth, among others, and encourages feelings such as envy and jealousy, in a reality where production relations are established only if there are inequalities in conditions^(8)^.

Thus, social competition is one of the constituent elements of capitalist sociability and “*is not only present in everyday reality and in the individual’s mentality, but also requires competitive behavior, under penalty of being considered ‘lazy,’ ‘abnormal,’ ‘failed,’ etc*.”^(8:31)^.

However, although issues related to the structure of society are at the root of student burnout, educational institutions tend to propose responses to them at the individual level, which can increase the burden of self-management of suffering^(7)^.

This tendency for institutional responses to focus on individuals was identified in a synthesis of evidence, in which the vast majority of studies, beyond the individual dimension, do not take into account aspects related to the socio-structural, collective, and institutional dimensions in the origins of students’ psychological suffering; therefore, they do not present collective responses to the problems identified^(1)^.

Therefore, it is possible to affirm that institutional responses in universities have been predominantly associated with encouraging self-care actions, at the individual level, as ways to alleviate the psychological distress of university students. These methods do not eliminate the distress, but allow students to return to their expected performance levels feeling somewhat more relieved, even if it does not take long for the distress to return. Based on the assumption that actions that focus only on the individual biopsychic sphere are insufficient to address the stresses of academic life, the elective course – “Coping with the wear and tear of university life” – was implemented, open to all undergraduate students at the University of São Paulo, to provide a space for discussion and listening about academic experiences, with a view to jointly building strategies to cope with wear and tear and its implications for university education. This study aimed to identify changes in students’ conceptions of the stresses of academic life and their causes and to work with students to develop practices and strategies for strengthening the academic experience during the course.

## METHOD

### Type of Study

This is a descriptive study with a qualitative approach based on participant research, the product of a master’s thesis. In this approach, the object-researcher relationship is replaced by the subject-subject relationship and, through dialogue, different knowledge is shared, producing an understanding of social reality and its transformation^(9)^.

### About the Course and the Research Subjects

The course was created with the aim of overcoming proposals for actions aimed at encouraging individual self-care in response to burnout and had the following objectives: a) To characterize the diverse youth with different profiles of social reproduction in contemporary times; b) To identify and analyze the potential for strengthening/burning out the university experience; c) To develop joint proposals to address the potential for burnout in the university experience.

The discipline was offered for the first time in November 2023, linked to the university’s Vice-dean’s Office, as an elective course for students entitled “Addressing burnout in the university experience.” It was taught at one of the 42 teaching units of the University of São Paulo, which has approximately 60,000 students. Although only 30 places were available for students from all undergraduate courses at this university, 776 students enrolled. It was decided to accept the selection made by the enrollment system, which chose the first thirty students who needed the credits from the course to graduate. Ultimately, nine students participated in the course and the research, as the others had interpreted that the course would be offered remotely, in the same way as most of the courses linked to the Office of the Vice-dean of Undergraduate Studies^1^, and were unable to participate in person.

The research subjects were the students who enrolled in this course and agreed to participate in the research. The invitation was made at the end of the course, after the grades were handed out, and the students chose to allow their records to be used as research analysis material, after giving their official consent by signing a Free and Informed Consent Form (FICF).

### Course Development

The course was developed by three professors from two different teaching units and a teaching assistant. Active strategies and methods were used to address the following topics: youth and contemporaneity, the university as a social organization, university experience, emancipatory education as a tool for improving the university experience, strategies for coping with burnout at the university, political participation, and social movements as tools for coping with burnout.

In order to identify improvements in students’ conceptions of the wear and tear of academic life and its causes, students were asked to answer open-ended questions at the beginning and end of the first meeting, in the form of pre- and post-tests, following the example of the study by Cordeiro et al.^(10)^. After explaining the purpose of this activity, students had about 40 minutes to answer the questions. At the first meeting, the questions were: – In your university experience, what strengths of academic life do you identify? – What would be the wear and tear and its causes? – What would be the ways to deal with this wear and tear? At the last meeting of the course, the questions from the first meeting were repeated, with the addition of the question: – Do you believe that participating in the course contributed to your learning process? In what ways?

The answers to these questions, referring to the beginning and end of the course, were analyzed to identify the course’s contribution to improving the concepts and perspectives of undergraduate students regarding the potential for stress/strengthening of the university experience and in the development of proposals to address it.

### Data Analysis

The students’ responses were analyzed using content analysis^(11)^. The analysis included the fragmentation of the records in a process of constructing units of meaning or themes, from which empirical categories were identified for the purpose of organizing and interpreting the content provided in the undergraduate students’ responses.

After organizing the content, the analysis was performed using concepts from the field of Collective Health.

### Ethical Aspects

The parent project was submitted to the Ethics and Research Committee of the USP School of Nursing, which issued a favorable final opinion, under number 6,749,093. The research met all the specifications of Resolution 466, dated December 12, 2012, which approves the guidelines and regulatory standards for research involving human beings. Participants were invited to read and sign the Free and Informed Consent Form (FICF) presented by the proponent of the parent project. All agreed to participate in the research.

## RESULTS

All participants were between 22 and 23 years old, three were female and six were male, and they were in different courses/schools: Computer Science, Audiovisual, Pharmacy, Geology, Electrical Engineering, and Nursing. Of these, six were in the last semester of the course and two were in the fourth semester, meaning they had already completed half of their undergraduate studies.

Below is a summary of the contents of the individual records of the undergraduate students made on the first day of class, called the pre-test, and on the last day, called the post-test.

Chart 1 presents the themes obtained from the answers to the question about the stresses that permeate academic life and the causes of these stresses.

**Chart 1:** Themes identified in the pre- and post-tests for the question “What are the stresses and their causes?” – São Paulo, SP, Brazil, 2024.

PRE-TEST Topics	POST-TEST Topics
Psychological illness in the academic community Competitive academic environment with high expectations Overwork and feelings of guilt Curriculum design with excessive content and no free areas Difficulty establishing interpersonal relationships Lack of teaching skills among professors and conflictual relationships between teachers and students Insufficient retention policies to meet the social profile of students Feeling of wasted time during commutes to the university, which could be used for studying Feelings of insecurity and uncertainty Feeling of identification and belonging undermined by prejudice Insufficient teaching staff	Psychological illness in the academic community The academic environment encouraging competitiveness and isolation Stimulation of the academic environment to overload activities Lack of teaching skills among teachers and conflictual relationships between teachers and students Inadequacy of retention policies to meet the social profile of students Feeling of not belonging Neoliberal values and the demand for academic productivity

It is important to note here that two themes caught the researchers’ attention, either because they were much more prevalent at the end of the course (post-test) compared to the records from the first day of class (pre-test), or because they were presented exclusively on the last day, denoting changes in students’ conceptions regarding the potential for wear and tear and strengthening of academic life, as well as proposals for addressing the wear and tear identified.

Thus, the **“Inadequacy of retention policies to meet the social profile of students”** was identified as one of the causes of wear and tear at both moments (pre- and post-test), but on the last day of class, this theme was brought up with greater intensity by the students. The theme **“Neoliberal values and the demand for academic productivity,”** in turn, was only mentioned in the post-test, and the students’ records made explicit the reproduction of neoliberal values in the university, such as competitiveness and isolation, as can be identified in the excerpt:

“The main reasons for these stresses are linked to the state of modern capitalist society. The university environment mirrors the external environment, which is why its change in recent years is noticeable. The university environment is increasingly productivity-oriented and competitive, with professors being forced to publish as many articles as possible so that the university can meet productivity targets to obtain government investment, prioritizing quantity over quality (...)”

Chart 2 contains the themes obtained from the records for the question about the strengths of academic life raised by the students.

**Chart 2:** Themes identified in the pre- and post-tests for the question “In your university experience, what strengths of academic life do you identify?” – São Paulo, SP, Brazil, 2024.

PRE-TEST Topics	POST-TEST Topics
Support networks formed by colleagues and family members Good relationships with teachers and mentors Institutional factors that contribute to student retention Taking advantage of undergraduate opportunities Curriculum organization that considers students' needs	Support networks formed by colleagues and family members (coupled with comments about the fact that people with similar social conditions tend to be closer to each other) Good relationships with teachers and mentors Institutional factors that contribute to student retention Taking advantage of undergraduate opportunities Curriculum organization that considers student needs Understanding of collective strength Understanding of structural issues (political and economic) that affect the university Understanding the causes of attrition

It appears that the last three themes were only found in the post-test, in which individual perspectives on strengthening were added to collective experiences. One of the themes added was **“Understanding collective strength”**, in which students point out that political participation and collective mobilization promote a more effective fight against the wear and tear present in the university environment.

Another theme pointed out exclusively in the posttest, **“Understanding of structural issues (political and economic) that affect the university,”** follows the logic that understanding the university as an institution that promotes wear and tear and strengthening, reproducing the values on which the society to which it belongs is based, can be a strengthening pillar for university students, as explained by one of the students on the last day of class:

“[The course] made me understand and realize that a certain ‘guilt’ I felt for not having done so many things, compared to some of my colleagues, has a root and origin that does not come from me. In addition, the course as a whole made me more aware of the problems in academic life today. Although they are always present, even in my routine, stopping to think and clarify the stresses faced by everyone was good for gaining more self-knowledge.”

The last theme, “Understanding the causes of suffering,” relates to an understanding of the roots of the stresses of university life, so that guilt is no longer exclusively the individual’s responsibility once the influence of the neoliberal values that the university reproduces is recognized.

The following are the themes related to the proposed actions and strategies for coping with the stresses identified by the participants at the beginning and end of the course (Chart 3).

**Chart 3:** Themes identified in the pre- and post-test for the question “What are some ways to cope with these stresses?” – São Paulo, SP, Brazil, 2024.

PRE-TEST Topics	POST-TEST Topics
Promotion of social spaces Involvement in extracurricular activities Reorientation of the curriculum Strategies to meet students’ educational needs Access to psychological support services More assertive retention policies Assertive strategies to combat prejudice and harassment Restructuring and pedagogical training of teaching staff Individual coping strategies (balancing time between studies and family life)	Promotion of spaces for interaction and coordination between students and staff, and between faculty and technical-administrative staff Improvement of training and involvement in extension activities Reorientation of the curriculum Strategies to meet students' educational needs Access to psychological support services Political and social engagement at the university Courses that promote reflection on the university Use of qualitative metrics to evaluate teaching at the university

At the end of the course, it became clear how much students had come to consider collective issues and political responses in the process of coping with burnout. From this perspective, the topic **“Political and social engagement at the university”** was the most cited as a way of coping with burnout, with six students expressing their views on how active participation in political and social movements, student collectives, actions that promote unity among the sectors that make up the university community, discussion groups, and student representation in departments, are of paramount importance for the construction of coping strategies.

“The way to cope with attrition is to unite the three sectors that make up the University and organize for political change, as well as to hold debates, meetings, and classes that aim to convey what attrition is and what its causes are, so that there is awareness and contributions from everyone.”

It is worth highlighting the statement about the need for participation by the three sectors (students, teaching staff, and technical-administrative staff) in discussion spaces. Student representation on university committees and collective student bodies was also cited as a strategy for political engagement.

Finally, the last theme/strategy identified only in the posttest analysis was the offering of **“Courses that promote space for reflection on the university”**, in which students indicated that the institutional creation of these spaces promotes the potential to address burnout.

## DISCUSSION

Among the potential sources of stress listed by students in the pre-test were those related to the material conditions of student existence and retention that directly interfere with the university experience^(12)^.

There are currently initiatives that expand programs that favor student retention. However, even with the existence of these programs, such as university restaurants and food scholarships, recognized as having the potential to strengthen academic life by students, they are not sufficient to respond effectively to the rapid transformation of the social reproduction profiles of students entering higher education institutions ^(4,12,13)^.

In addition to the need for resources to support university retention policies, it is also necessary to strengthen support networks and interpersonal relationships. Lack of family support and difficulty in establishing interpersonal relationships are elements that undermine the university experience^(9)^. Belonging to groups, family support, and friendships contribute to coping with difficulties, as students feel they belong to collectives^(4,12,14)^.

From the perspective of interpersonal relationships, it is important to highlight that the incentivized competition in the academic environment weakens bonds and support networks, replacing supportive relationships with competitive ones.

The “culture of competitiveness” is one of the negative aspects of the university student experience^(12)^. The intense competitiveness observed in the academic environment is considered a consequence of the reproduction of neoliberal ideals in the university^(13,15)^.

Neoliberal society, in general, increasingly places all responsibility for success or failure in all areas of life on the individual. This logic, reinforced by the process of individualization^(16)^, leads to the breakdown of collective bonds, as social relationships become emptier and less meaningful. Thus, it is solely up to the individual to dedicate themselves sufficiently to achieve their goals^(15)^.

The difficulty of dealing with this excessive responsibility, with the scarcity of opportunities and with the lack of support from institutions leads to feelings of helplessness and guilt, intensifying the suffering of not reaching the level imposed as ideal^(7,13,15)^.

At the university, this context is aggravated by “academic productivism,” a phenomenon structured on the logic of excessive valorization of the quantity of scientific and academic production to the detriment of its quality^(17,18)^.

The university is immersed in the social relations of the hegemonic mode of production; therefore, it is governed by capitalist sociability, in which elements such as the commodification and bureaucratization of social relations are constitutive. The university no longer directs its practices of training workers and conducting research to respond to the needs of society, but rather gives priority to the needs of the market and the production of goods. In this way, the public university assumes its character as an operational university, which prioritizes individualization, performance, and efficiency^(19)^.

The students participating in this study identified the lack of teaching skills among professors as one of the potential causes of academic burnout, which directly impacts the quality of teaching and the willingness of professors to teach, in addition to being a major source of stress for undergraduate students. The difficulty of transitioning from theoretical to practical content, outdated pedagogical strategies, lack of attention given to students, and lack of knowledge updating lead to dissatisfaction with the course^(20)^.

An aggravating factor is that there is also a lack of dialogue and listening between teachers and students about the processes of constructing the curriculum^(20)^. The intense workload or fulltime schedule, the accumulation of mandatory activities, and the workload at universities are causes of stress^(4)^.

Thus, it appears that stress in academic life is related to issues beyond the individual difficulties of students and concerns the process of adaptation to academic life. They concern structural elements that involve, among other aspects, the failure to meet the distinct needs of students determined by their social reproduction profiles, the hierarchical relationship between teachers and students, and the organization of the curriculum.

On the other hand, when universities identify manifestations of wear and tear in psychological distress, they offer care strategies that are predominantly curative, i.e., symptom-oriented, and actions focused on self-care^(1,14)^. The phenomenon is homogenized and individualized^(6,17)^.

It is necessary to devise collective strategies that enable institutions to address the psychological distress of these young university students^(1)^, as indicated by the students themselves. Political and social engagement and the organization of individuals into groups around a common goal/theme represent a way to combat the process of individualization. In order for these spaces to enable the development of tools to cope with stress, the collective dimensions of the origins of suffering^(7)^ must be considered, as recorded in the post-test.

It is important that collective actions be implemented in the academic environment, as they have the potential to strengthen and transform. Collective spaces promote health and challenge behaviors that make students ill^(21)^. The meetings provide the power to act and, consequently, to cope with burnout.

## CONCLUSION

Based on the students’ records, it was found that the course provoked reflections on how political and social participation, in addition to the organization of subjects into groups, is an important potential for strengthening the academic experience, permeated by the stressing and ill-favoring logic of capitalist sociability.

The course helped students improve their understanding of the wear and tear of university life and its causes, as well as the potential for strengthening during undergraduate studies as a way to counteract the potential for stress.

From this perspective, improvements can be identified with regard to the collective perspective of confrontation, making explicit the need to act on the roots of wear and tear through active political participation.

The present study was limited by the number of participants. It is important that new studies be conducted based on future offerings of the course with other selection criteria to increase the number of students.

It is believed that the course should be offered continuously, providing space for reflection, discussion, and facilitation of collective actions to strengthen academic life.

## Data Availability

The entire dataset supporting the results of this study is available upon request to the corresponding author.
