# Supplementary material for: Confrontations and wear and tear of academic life: a proposal for an undergraduate course
Source: Rev Esc Enferm USP. 2026 Jan 16;59:e20250115. doi: 10.1590/1980-220X-REEUSP-2025-0115en (PMC12836612; doi:10.1590/1980-220X-REEUSP-2025-0115en)
Supplement: Translation

# Translation: Enfrentamentos e desgastes da vida acadêmica: uma proposta de disciplina para a graduação^*^

**DOI:** 10.1590/1980-220X-REEUSP-2025-0115pt

**Authors:** Ricardo Santoro, Carla Andrea Trapé, Célia Maria Sivalli Campos, Ana Claudia Camargo Gonçalves Germani, Iasmin Cordeiro de Oliveira, Paulo de Melo Guimarães

**Affiliations:** 1 Universidade de São Paulo, Escola de Enfermagem, Departamento de Enfermagem em Saúde Coletiva, São Paulo, SP, Brasil.; 2 Universidade de São Paulo, Faculdade de Medicina, Departamento de Medicina Preventiva, São Paulo, SP, Brasil.

**Keywords:** Universidades, Estresse Psicológico, Políticas, Pesquisa Participativa Baseada na Comunidade

## Abstract

**Objetivo:**

Identificar as contribuições da Disciplina “Enfrentamento dos desgastes da vivência universitária” para propiciar a construção conjunta de estratégias pelos estudantes para o fortalecimento da vivência acadêmica.

**Método:**

Trata-se de um estudo descritivo com abordagem qualitativa pautado na pesquisa-participante, produto de uma dissertação de mestrado. Foi solicitado aos estudantes que respondessem questões abertas relacionadas às causas dos desgastes da vida acadêmica e estratégias de enfrentamento aplicadas no primeiro e último dia de aula.

**Resultados e Discussão:**

Participaram da disciplina 9 estudantes de graduação. Ao final da disciplina, as concepções dos estudantes sobre os desgastes da vida acadêmica e suas causas passaram a incluir a dimensão estrutural da reprodução dos valores neoliberais na universidade e as estratégias de enfrentamento se concentraram nas relacionadas aos movimentos estudantis e participação de coletivos.

**Conclusão:**

A disciplina contribuiu para a compreensão das raízes dos desgastes da vivência acadêmica dos estudantes e elaboração de propostas de enfrentamento coletivas.

## INTRODUÇÃO

A vivência universitária tem se caracterizado por sobrecarga de produção, em contexto marcado pela necessidade de respostas a variadas demandas, que geram competitividade e inseguranças no futuro, produzindo desgastes manifestados como sofrimento psíquico^(1)^.

Pesquisa realizada com 136 mil graduandos identificou que 80% desses estudantes sinalizaram ansiedade, sensação de desânimo e falta de vontade, insônia, desamparo no meio familiar, solidão, transtornos alimentares, síndrome do pânico e pensamento suicida^(2)^. Estudo realizado com graduandos da área da saúde encontrou resultados preocupantes; 70,7% apresentavam manifestações de sofrimento psíquico, com chances 2,4 vezes maiores de ocorrerem em estudantes de famílias de grupos sociais com condições mais instáveis nas condições de trabalho e vida^(3)^.

Este estudo ancora-se nas bases teórico-metodológicas do campo da Saúde Coletiva, que define os resultados do processo saúde-doença como resultado do embate entre potenciais de fortalecimento e desgaste advindos dos perfis de reprodução social característicos das distintas classes sociais^(4)^.

Parte-se do pressuposto de que os desgastes são vivenciados desigualmente pelos estudantes de diferentes condições sociais^(5)^ e, considerando-se que a maior parte dos graduandos é jovem, os diferentes perfis de reprodução social determinam os distintos perfis de juventude universitária, com heterogêneas condições de trabalho, vida e saúde^(5)^. Sendo assim, compreender a juventude a partir da reprodução social, identificando potenciais de fortalecimento e desgaste possibilita a superação de teorias hegemônicas do processo saúde-doença^(6)^ que desconsideram a relação entre inserção de classe e condições de saúde da população.

Nessa perspectiva, é importante considerar que os jovens de distintas classes sociais vivenciam desigualmente o contexto social e, consequentemente, o universitário^(6)^. O sofrimento é a expressão no corpo do que é produzido na sociedade neoliberal capitalista e reproduzido nas universidades, afetando os graduandos e potencializando os desgastes gerados pelas pressões decorrentes do alto nível de competitividade, desesperança na inserção no mercado de trabalho, exigências pelo aumento da produtividade acadêmica, entre outros^(7)^.

A sociabilidade capitalista reforça valores associados a sucesso individual, riqueza, entre outros e incentiva sentimentos como inveja, ciúmes, numa realidade em que as relações de produção se estabelecem somente se houver desigualdades de condições^(8)^.

Assim, a competição social é um dos elementos constitutivos da sociabilidade capitalista e *“não só está presente na realidade cotidiana e na mentalidade do indivíduo, como exige dele um comportamento competitivo, sob pena de ser considerado “preguiçoso”, “anormal”, “fracassado”, etc”* ^(8:31)^.

No entanto, embora sejam questões relacionadas à estrutura da sociedade que estão nas raízes dos desgastes dos estudantes, as instituições de ensino tendem a propor respostas a eles no âmbito individual, o que pode estimular o aumento do peso que representa a autoadministração do sofrimento^(7)^.

Essa tendência de respostas institucionais focalizadas nos indivíduos foi identificada em síntese de evidências, em que a grande maioria dos estudos, para além da dimensão individual, não tomam em conta aspectos ligados às dimensões socioes-truturais, coletivas e institucionais nas origens do sofrimento psíquico de estudantes; logo, não apresentam respostas coletivas aos problemas identificados^(1)^.

Portanto, é possível afirmar que as respostas institucionais em universidades têm sido preponderantemente associadas ao estímulo a ações de autocuidado, na dimensão individual, como formas de aliviar o sofrimento psíquico de universitários, formas essas que não remitem o sofrimento, mas permitem que os estudantes retornem um pouco mais aliviados para o desempenho do que é esperado, mesmo que não demore muito tempo para o sofrimento retornar. Partindo-se do pressuposto de que ações que incidam apenas no âmbito biopsíquico individual são insuficientes para enfrentar os desgastes da vida acadêmica, foi implementada a disciplina optativa – “Enfrentamento dos desgastes da vivência universitária” – aberta a todos os estudantes de graduação da Universidade de São Paulo, para propiciar espaço de discussão e escuta a respeito das vivências acadêmicas, com vistas à construção conjunta de estratégias para enfrentamento de desgastes e de suas implicações na formação universitária. O presente estudo se propôs a identificar mudanças de concepções dos estudantes sobre os desgastes da vida acadêmica e suas causas e elaborar em conjunto com os estudantes a proposição de práticas e estratégias para o fortalecimento da vivência acadêmica no decorrer da disciplina.

## MÉTODO

### Tipo de Estudo

Trata-se de um estudo descritivo com abordagem qualitativa pautado na pesquisa-participante, produto de uma dissertação de mestrado. Nessa abordagem, a relação objeto-investigador é substituída pela relação sujeito-sujeito e, por meio do diálogo, diferentes conhecimentos são compartilhados produzindo a compreensão da realidade social e sua transformação^(9)^.

### Sobre a Disciplina e os Sujeitos da Pesquisa

A disciplina foi criada com a finalidade de superar as propostas de ações voltadas ao incentivo do autocuidado individual, em resposta aos desgastes e tomou por objetivos: a) Caracterizar as diversas juventudes com distintos perfis de reprodução social na contemporaneidade; b) Identificar e analisar potenciais de fortalecimento/desgaste da vivência universitária; c) Elaborar propostas conjuntas para o enfrentamento dos potenciais de desgaste da vivência universitária.

A disciplina foi oferecida pela primeira vez em novembro de 2023, vinculada à Pró-reitoria da universidade, na modalidade optativa para os estudantes e intitulada “Enfrentamento dos desgastes da vivência universitária”. Foi ministrada em uma das 42 unidades de ensino da Universidade de São Paulo que conta com cerca de 60.000 alunos. Embora tenham sido disponibilizadas apenas 30 vagas para estudantes de todos os cursos de graduação desta universidade, 776 estudantes se inscreveram. Optou-se por aceitar a seleção do sistema de matrículas que escolheu os primeiros trinta alunos que dependiam dos créditos da disciplina para se formarem. Por fim participaram da disciplina e da pesquisa nove estudantes, pois os demais haviam interpretado que o oferecimento seria no formato remoto, da mesma forma que a maior parte das disciplinas vinculadas à Pró-reitoria de graduação^1^, e não puderam participar presencialmente.

Os sujeitos da pesquisa foram os estudantes que se matricularam nessa disciplina e que aceitaram participar da pesquisa. O convite foi feito no final da disciplina, após a entrega das notas e os estudantes optaram por permitir que seus registros fossem utilizados como material de análise da pesquisa, após consentimento oficializado com a assinatura em Termo de Consentimento Livre e Esclarecido (TCLE).

### Desenvolvimento da Disciplina

A disciplina foi desenvolvida por três docentes de duas unidades de ensino distintas e um monitor Foram utilizadas estratégias e métodos ativos, para abordagem dos temas: juventudes e contemporaneidade, a Universidade como organização social, vivência universitária, educação emancipatória como instrumento de aprimoramento da vivência universitária, estratégias de enfrentamento dos desgastes na universidade, participação política e movimentos sociais como ferramentas para o enfrentamento de desgastes.

Com a finalidade de se identificar aprimoramentos nas concepções dos estudantes sobre os desgastes da vida acadêmica e suas causas, foi solicitado que os estudantes respondessem, no início do primeiro encontro e no último, questões abertas, nos moldes de pré e pós-teste, a exemplo do estudo de Cordeiro et al.^(10)^. Após a explicação do objetivo dessa atividade os estudantes dispuseram de cerca de 40 minutos para responder as perguntas. No primeiro encontro as questões foram: – Na sua vivência universitária, quais fortalecimentos da vida acadêmica você identifica? – Quais seriam os desgastes e suas causas? – Quais seriam as formas de enfrentar esses desgastes? No último encontro da disciplina se repetiram as perguntas do primeiro encontro, acrescidas da questão: – Você acredita que participar da disciplina trouxe contribuições para seu processo de formação? Em que aspectos?

As respostas a essas perguntas, referentes ao início e final da disciplina, foram analisadas, para identificar a contribuição da disciplina no aprimoramento de conceitos e perspectivas dos graduandos a respeito dos potenciais de desgaste/fortalecimento da vivência universitária e na elaboração de propostas de enfrentamento.

### Análise dos Dados

As respostas dos estudantes foram analisadas pelo método da análise de conteúdo^(11)^. A análise contemplou a fragmentação dos registros num processo de construção de unidades de significação ou temas, a partir das quais identificou-se as categorias empíricas, com a finalidade de organização e interpretação do conteúdo trazido nas respostas dos graduandos.

Após a organização do conteúdo, a análise foi feita utilizando os conceitos do campo da Saúde Coletiva.

### Aspectos Éticos

O projeto matriz foi submetido ao Comitê de Ética e Pesquisa da Escola de Enfermagem da USP que emitiu parecer final favorável, sob o número 6.749.093. A pesquisa atendeu a todas as especificações da Resolução 466, de 12 de dezembro de 2012, que aprova as diretrizes e normas regulamentadoras de pesquisas envolvendo seres humanos. Os participantes foram convidados a ler e assinar o Termo de Consentimento Livre e Esclarecido (TCLE) que foi apresentado pelo proponente do projeto matriz. Todos concordaram em participar da pesquisa.

## RESULTADOS

Todos os participantes tinham entre 22 e 23 anos de idade, três do sexo feminino e seis do sexo masculino e estavam em cursos/Escolas diferentes: Ciência da Computação, Audiovisual, Farmácia, Geologia, Engenharia Elétrica e Enfermagem. Destes, seis estavam no último semestre do curso e dois no quarto semestre, ou seja, já haviam cursado metade da graduação.

A seguir serão apresentadas sínteses dos conteúdos dos registros individuais dos graduandos realizados no primeiro dia de aula, denominado de pré-teste, e no último encontro, denominado pós-teste.

O Quadro 1 apresenta os temas obtidos a partir das respostas à pergunta sobre quais os desgastes que permeiam a vida acadêmica e quais as causas desses desgastes.

**Quadro 1:** Temas identificados no pré e pós teste para a pergunta “Quais seriam os desgastes e suas causas?” – São Paulo, SP, Brasil, 2024.

Temas PRÉ-TESTE	Temas PÓS-TESTE
Adoecimento psicológico da comunidade acadêmica Ambiente acadêmico competitivo e de altas expectativas Sobrecarga e sentimento de culpa Formulação da matriz curricular com sobrecarga de conteúdos e sem áreas livres Dificuldade de estabelecer relações interpessoais Falta de didática dos professores e relações conflituosas entre docentes e discentes Insuficiência das políticas de permanência para atender ao perfil social dos estudantes Sensação de desperdício de tempo durante a locomoção para a universidade, que poderia ser utilizado para estudo Sentimentos de insegurança e incerteza Sentimento de identificação e pertencimento prejudicado pelo preconceito Quadro docente insuficiente	Adoecimento psicológico da comunidade acadêmica Estímulo do ambiente acadêmico à competitividade e isolamento Estímulo do ambiente acadêmico à sobrecarga de atividades Falta de didática dos professores e relações conflituosas entre docentes e discentes Insuficiência das políticas de permanência para atender ao perfil social dos estudantes Sensação de não pertencimento Valores neoliberais e a exigência do produtivismo acadêmico

É importante ressaltar aqui que dois temas chamaram a atenção dos pesquisadores ou porque estiveram muito mais presentes no final da disciplina (pós-teste) em comparação aos registros do primeiro dia de aula (pré-teste), ou ainda porque foram apresentados exclusivamente no último dia denotando mudanças nas concepções dos estudantes a respeito dos potenciais de desgastes e fortalecimentos da vida acadêmica, bem como propostas de enfrentamento dos desgastes identificados.

Assim, a **“Insuficiência das políticas de permanência para atender ao perfil social dos estudantes”** foi identificada como uma das causas dos desgastes em ambos os momentos (pré e pós teste), mas no último dia de aula esse tema foi trazido com mais intensidade pelos estudantes. O tema **“Valores neoliberais e a exigência do produtivismo acadêmico”,** por sua vez, foi citado apenas no pós-teste e os registros dos estudantes explicitaram a reprodução dos valores neoliberais na universidade, como competitividade e isolamento, como pode ser identificado no trecho:

“Os principais motivos desses desgastes estão atrelados ao estado da sociedade moderna capitalista. O ambiente universitário se espelha no ambiente externo, por isso é perceptível sua mudança durante os últimos anos. O ambiente universitário está cada vez mais produtivista e competitivo, os professores são obrigados a publicar o maior número possível de artigos para que a universidade consiga bater as metas de produtividade para conseguir investimento do governo, priorizando quantidade sobre qualidade (...)”

O Quadro 2 contém os temas obtidos a partir dos registros para a pergunta sobre os fortalecimentos da vida acadêmica levantados pelos estudantes.

**Quadro 2:** Temas identificados no pré e pós teste para a pergunta “Na sua vivência universitária, quais fortalecimentos da vida acadêmica que você identifica?” – São Paulo, SP, Brasil, 2024.

Temas PRÉ-TESTE	Temas PÓS-TESTE
Redes de apoio formadas por colegas e familiares Boa relação com docentes e mentores Fatores institucionais que contribuem para a permanência estudantil Aproveitamento das oportunidades da graduação Organização do currículo que considera as necessidades dos estudantes	Redes de apoio formadas por colegas e familiares (acrescidas de falas sobre o fato de que a proximidade é maior entre pessoas com as mesmas condições sociais) Boa relação com docentes e mentores Fatores institucionais que contribuem para a permanência estudantil Aproveitamento das oportunidades da graduação Organização do currículo que considera as necessidades dos estudantes Compreensão da força coletiva Compreensão sobre questões estruturais (políticas e econômicas) que atravessam a universidade Compreensão das causas dos desgastes

Verifica-se que os últimos três temas foram encontrados somente no pós-teste, nas quais perspectivas de fortalecimento individuais foram somadas a experiências coletivas. Um dos temas adicionados foi a **“Compreensão da força coletiva”**, no qual os estudantes apontam que a participação política e mobilização coletiva promovem um combate mais efetivo dos desgastes presentes no ambiente universitário.

Outro tema apontado exclusivamente no pós-teste, **“Compreensão sobre questões estruturais (políticas e econômicas) que atravessam a universidade”,** segue a lógica de que a compreensão da universidade como uma instituição promotora de desgastes e fortalecimentos, reprodutora de valores nos quais a sociedade à qual pertence se baseia, pode se configurar como um ponto de fortalecimento para os universitários como explicitado por um dos estudantes no último dia de aula:

“[a disciplina] me fez entender e perceber que uma certa “culpa” que eu sentia por não ter feito tantas coisas, comparado a alguns colegas, tem uma raiz e origem que não vem de mim. Além disso, a disciplina como um todo me fez mais consciente dos problemas na vivência acadêmica hoje em dia. Apesar de eles sempre estarem presentes, até na minha rotina, parar pra pensar e esclarecer quais são os desgastes enfrentados por todos foi bom para ter adquirir mais autoconhecimento.”

O último tema **“Compreensão das causas do sofrimento”,** relaciona-se a uma compreensão acerca das raízes dos desgastes da vivência universitária, de modo que a culpa deixa de ser exclusivamente do indivíduo a partir do reconhecimento da influência dos valores neoliberais que a universidade reproduz.

A seguir apresentam-se os temas relacionados às propostas de ações e estratégias de enfrentamento dos desgastes identificadas pelos participantes no início e no final da disciplina (Quadro 3).

**Quadro 3:** Temas identificados no pré e pós teste para a pergunta “Quais seriam as formas de enfrentar esses desgastes?” – São Paulo, SP, Brasil, 2024.

Temas PRÉ-TESTE	Temas PÓS-TESTE
Promoção de espaços de convivência Envolvimento em atividades extracurriculares Reorientação da matriz curricular Estratégias de atendimento às necessidades pedagógicas dos estudantes Acesso a serviços de apoio psicológico Política de permanência mais assertivas Estratégias assertivas de combate ao preconceito e o assédio Recomposição e formação pedagógica do corpo docente Estratégias individuais como enfrentamento (organização do tempo entre estudos e convivência familiar)	Promoção de espaços de convivência e articulação entre discentes e servidores e entre docentes e técnico-administrativos Aprimoramento da formação e envolvimento em atividades de extensão Reorientação da matriz curricular Estratégias de atendimento às necessidades pedagógicas dos estudantes Acesso a serviço de apoio psicológico Engajamento político e social na universidade Disciplinas que promovam espaço de reflexão sobre a universidade Uso de métricas qualitativas para avaliação do ensino na universidade

Ao final da disciplina ficou explícito o quanto os estudantes passaram a considerar questões coletivas e respostas políticas no processo de enfrentamento dos desgastes. Nessa perspectiva, o tema **“Engajamento político e social na universidade”** foi o mais citado como forma de enfrentamento de desgastes, com seis estudantes se manifestando sobre como a participação ativa em movimentos políticos e sociais, em coletivos estudantis, em ações que promovem a união dos setores que compõem a comunidade universitária, em rodas de conversa, em representação discente em departamentos, são de suma importância para a construção de estratégias de enfrentamento.

“A forma de enfrentamento dos desgastes é a união dos três setores que compõem a Universidade e a organização para mudanças políticas, também a realização de debates, reuniões e disciplinas que visem transferir quais são os desgastes e quais suas causas, para que haja consciência e contribuições de todos.”

Ressalta-se a afirmação sobre a necessidade de participação dos três setores (discentes, servidores docentes e servidores técnicos-administrativos) em espaços de discussão. A representação discente em comissões e entidades coletivas estudantis da universidade também foi citada como estratégia de engajamento político.

Por fim, o último tema/estratégia identificado somente na análise do pós-teste foi o oferecimento de **“Disciplinas que promovam espaço de reflexão sobre a universidade”**, na qual os discentes sinalizaram que a criação institucional desses espaços promove um potencial de enfrentamento aos desgastes.

## DISCUSSÃO

Dentre os potenciais de desgaste elencados pelos estudantes, registrados no pré-teste, foram relacionadas às condições materiais de existência e permanência estudantil que interferem diretamente na vivência universitária^(12)^.

Atualmente existem iniciativas que ampliam os programas que favorecem a permanência estudantil. Entretanto, mesmo com a existência desses programas, como restaurantes universitários e bolsas-alimentação, reconhecidos como potenciais de fortalecimento da vida acadêmica pelos estudantes, não são suficientes para responder de maneira eficaz à rápida transformação dos perfis de reprodução social dos estudantes que ingressam nas instituições de ensino superior ^(4,12,13)^.

Além da necessidade de recursos que apoiem políticas de permanência na universidade, é necessário também o fortalecimento de redes de apoio e de relações interpessoais. A falta de apoio familiar e a dificuldade em estabelecer relacionamentos interpessoais são elementos que prejudicam a vivência universitária^(9)^. O pertencimento a grupos, o apoio familiar e as amizades contribuem para o enfrentamento das dificuldades, já que se sentem pertencentes a coletivos^(4,12,14)^.

Na perspectiva das relações interpessoais é importante destacar que a competição estimulada no ambiente acadêmico enfraquece os laços e as redes de apoio, substituindo as relações solidárias por relações competitivas.

A “cultura de competitividade” é um dos aspectos negativos da experiência dos estudantes universitários^(12)^. A intensa competitividade observada no meio acadêmico é considerada uma consequência da reprodução de ideais neoliberais na universidade^(13,15)^.

A sociedade neoliberal, de modo geral, cada vez mais coloca sobre o indivíduo toda a responsabilidade pelo seu sucesso ou fracasso em todas as áreas de sua vida. Essa lógica, reforçada pelo processo de individualização^(16)^, acarreta a quebra de vínculos coletivos, já que as relações sociais se tornam mais vazias e com menos sentido. Desse modo, cabe exclusivamente ao sujeito o dever de dedicar-se o suficiente para alcançar os seus objetivos^(15)^.

A dificuldade de lidar com essa responsabilidade excessiva, com a escassez de oportunidades e com o desamparo por parte das instituições leva ao sentimento de impotência e culpa, potencializando o sofrimento por não alcançar o patamar imposto como ideal^(7,13,15)^.

Na universidade esse contexto é agravado pelo “produtivismo acadêmico”, fenômeno que se estrutura na lógica da excessiva valorização da quantidade da produção científico-acadêmica em detrimento de sua qualidade^(17,18)^.

A Universidade está imersa no conjunto das relações sociais do modo de produção hegemônico; portanto, é regida pela sociabilidade capitalista, em que são constitutivos elementos como a mercantilização e a burocratização das relações sociais. A Universidade deixa de direcionar suas práticas de formação de trabalhadores e de pesquisas para responder às necessidades da sociedade, para dar prioridade às necessidades do mercado, a produção de mercadorias. Dessa forma, a universidade pública assume o seu caráter de universidade operacional, que prioriza a individualização, o desempenho e eficácia^(19)^.

Os estudantes participantes deste estudo identificaram como um dos potenciais de desgaste da vida acadêmica a falta de didática dos professores, o que impacta diretamente na qualidade do ensino e na disponibilidade do docente para ser professor, além de ser um grande gerador de desgaste para os graduandos. A dificuldade de realizar a transição do conteúdo teórico para o prático, estratégias pedagógicas ultrapassadas, falta de atenção dada aos estudantes, falta de atualização do conhecimento, trazem descontentamento em relação ao curso^(20)^.

Um agravante é que há também falta de diálogo e escuta dos docentes e discentes sobre os processos de construção da matriz curricular^(20)^. A carga horária intensa ou com período integral, o acúmulo de atividades obrigatórias e a carga horária das universidades são causadores de desgastes^(4)^.

Assim, verifica-se que os desgastes na vida acadêmica se relacionam a questões para além de dificuldades individuais dos estudantes e são concernentes ao processo de adaptação à vida acadêmica. Dizem respeito a elementos estruturais que envolvem, entre outros aspectos, o não atendimento das necessidades distintas dos estudantes determinadas pelos seus perfis de reprodução social, à relação hierarquizada entre docentes e discentes e à organização da matriz curricular.

Por outro lado, as universidades, ao identificarem as manifestações dos desgastes em sofrimento psíquico, ofertam estratégias de cuidado, predominantemente curativas, ou seja, voltadas a sintomas, e ações centradas no autocuidado^(1,14)^. O fenômeno é homogeneizado e individualizado^(6,17)^.

É necessário que sejam pensadas estratégias coletivas que possibilitem o enfrentamento institucional do sofrimento psíquico desses jovens universitários^(1)^, como sinalizado pelos estudantes. O engajamento político e social e a organização dos sujeitos em grupos, em torno de um objetivo/tema comum, representa uma forma de combater o processo de individualização. Para que esses espaços possibilitem o desenvolvimento de instrumentos para o enfrentamento de desgastes, devem ser consideradas as dimensões coletivas das origens de sofrimento^(7)^, como foi registrado no pós-teste.

É importante que ações coletivas sejam implementadas no ambiente acadêmico, já que são potenciais de fortalecimento e de transformação. Os espaços coletivos promovem saúde e contestam as condutas que adoecem os estudantes^(21)^. Os encontros proporcionam a potência de agir e, consequentemente, de enfrentar desgastes.

## CONCLUSÃO

A partir dos registros dos estudantes, verificou-se que a disciplina provocou reflexões sobre como a participação política e social, além da organização dos sujeitos em grupos, é um importante potencial de fortalecimento para a vivência acadêmica, permeada pela lógica desgastante e adoecedora da sociabilidade capitalista.

A disciplina contribuiu para que os estudantes aprimorassem concepções acerca dos desgastes da vivência universitária e suas causas, sobre os potenciais de fortalecimento durante a graduação como formas de propor embate aos potenciais de desgaste.

Nessa perspectiva, identificaram-se aprimoramentos no que diz respeito à perspectiva coletiva do enfrentamento, explicitando-se a necessidade de agir sobre as raízes dos desgastes, por meio de uma participação política ativa.

O presente trabalho teve como limitação o número de participantes. É importante que novos estudos sejam feitos a partir de futuros oferecimentos da disciplina com outros critérios de seleção para ampliar o número de estudantes.

Acredita-se que a disciplina deva ser oferecida continuamente, propiciando espaço de reflexão, discussão e facilitação de ações coletivas de fortalecimento da vida acadêmica.

## Data Availability

Todo o conjunto de dados que dá suporte aos resultados deste estudo está disponível mediante solicitação ao autor correspondente.

# Translation: Afrontamientos y desgastes de la vida académica: una propuesta de asignatura para la licenciatura^*^

**DOI:** 10.1590/1980-220X-REEUSP-2025-0115es

**Authors:** Ricardo Santoro, Carla Andrea Trapé, Célia Maria Sivalli Campos, Ana Claudia Camargo Gonçalves Germani, Iasmin Cordeiro de Oliveira, Paulo de Melo Guimarães

**Affiliations:** 1 Universidade de São Paulo, Escola de Enfermagem, Departamento de Enfermagem em Saúde Coletiva, São Paulo, SP, Brasil.; 2 Universidade de São Paulo, Faculdade de Medicina, Departamento de Medicina Preventiva, São Paulo, SP, Brasil.

**Keywords:** Universidades, Estrés Psicológico, Políticas, Investigación Participativa Basada en la Comunidad

## Abstract

**Objetivo:**

Identificar las contribuciones de la asignatura “Afrontamiento del desgaste de la vida universitaria” para propiciar la construcción conjunta de estrategias por parte de los estudiantes para fortalecer la experiencia académica.

**Método:**

Se trata de un estudio descriptivo con enfoque cualitativo basado en la investigación participante, producto de una tesis de maestría. Se pidió a los estudiantes que respondieran preguntas abiertas relacionadas con las causas del desgaste de la vida académica y las estrategias de afrontamiento aplicadas en el primer y último día de clase.

**Resultados y Discusión:**

Participaron en la asignatura nueve estudiantes de licenciatura. Al final de la asignatura, las concepciones de los estudiantes sobre el desgaste de la vida académica y sus causas pasaron a incluir la dimensión estructural de la reproducción de los valores neoliberales en la universidad, y las estrategias de afrontamiento se centraron en las relacionadas con los movimientos estudiantiles y la participación de colectivos.

**Conclusión:**

La asignatura contribuyó a la comprensión de las raíces del desgaste de la experiencia académica de los estudiantes y a la elaboración de propuestas de afrontamiento colectivo.

## INTRODUCCIÓN

La experiencia universitaria se ha caracterizado por una sobrecarga de producción, en un contexto marcado por la necesidad de responder a diversas demandas, lo que genera competitividad e inseguridad sobre el futuro, produciendo un desgaste que se manifiesta en forma de sufrimiento psíquico^(1)^.

Una investigación realizada con 136 000 estudiantes universitarios identificó que el 80 % de ellos manifestaban ansiedad, sensación de desánimo y falta de voluntad, insomnio, desamparo en el entorno familiar, soledad, trastornos alimentarios, síndrome de pánico y pensamientos suicidas^(2)^. Un estudio realizado con estudiantes de la carrera de salud encontró resultados preocupantes: el 70.7 % presentaba manifestaciones de sufrimiento psíquico, con probabilidades 2.4 veces mayores de ocurrir en estudiantes de familias de grupos sociales con condiciones más inestables en cuanto a trabajo y vida^(3)^.

Este estudio se basa en los fundamentos teóricometodológicos del campo de la salud colectiva, que define los resultados del proceso salud-enfermedad como el resultado del choque entre los potenciales de fortalecimiento y desgaste derivados de los perfiles de reproducción social característicos de las distintas clases sociales^(4)^.

Se parte del supuesto de que el desgaste se experimenta de manera desigual por los estudiantes de diferentes condiciones sociales^(5)^ y, considerando que la mayoría de los estudiantes universitarios son jóvenes, los diferentes perfiles de reproducción social determinan los distintos perfiles de la juventud universitaria, con condiciones heterogéneas de trabajo, vida y salud^(5)^. Por lo tanto, comprender la juventud a partir de la reproducción social, identificando los potenciales de fortalecimiento y desgaste, permite superar las teorías hegemónicas del proceso salud-enfermedad^(6)^ que descartan la relación entre la inserción de clase y las condiciones de salud de la población.

Desde esta perspectiva, es importante considerar que los jóvenes de diferentes clases sociales viven de manera desigual el contexto social y, en consecuencia, el universitario^(6)^. El sufrimiento es la expresión en el cuerpo de lo que se produce en la sociedad neoliberal capitalista y se reproduce en las universidades, afectando a los estudiantes universitarios y potenciando el desgaste generado por las presiones derivadas del alto nivel de competitividad, la desesperanza en la inserción en el mercado laboral, las exigencias de aumento de la productividad académica, entre otras^(7)^.

La sociabilidad capitalista refuerza valores asociados al éxito individual, la riqueza, entre otros, e incentiva sentimientos como la envidia y los celos, en una realidad en la que las relaciones de producción solo se establecen si existen desigualdades de condiciones^(8)^.

Así, la competencia social es uno de los elementos constitutivos de la sociabilidad capitalista y “*no solo está presente en la realidad cotidiana y en la mentalidad del individuo, sino que le exige un comportamiento competitivo, so pena de ser considerado “perezoso”, “anormal”, “fracasado”, etc.”* ^(*8:*31)^.

Sin embargo, aunque son cuestiones relacionadas con la estructura de la sociedad las que están en el origen del desgaste de los estudiantes, las instituciones educativas tienden a proponer respuestas a nivel individual, lo que puede estimular el aumento del peso que representa la autogestión del sufrimiento^(7)^.

Esta tendencia de respuestas institucionales centradas en los individuos se identificó en una síntesis de evidencias, en la que la gran mayoría de los estudios, más allá de la dimensión individual, no tienen en cuenta aspectos relacionados con las dimensiones socioestructurales, colectivas e institucionales en los orígenes del sufrimiento psíquico de los estudiantes; por lo tanto, no presentan respuestas colectivas a los problemas identificados^(1)^.

Por lo tanto, se puede afirmar que las respuestas institucionales en las universidades se han asociado predominantemente con el estímulo de acciones de autocuidado, en la dimensión individual, como formas de aliviar el sufrimiento psíquico de los universitarios, formas que no remiten el sufrimiento, pero permiten que los estudiantes regresen un poco más aliviados para el desempeño de lo que se espera, aunque no tarde mucho tiempo en volver el sufrimiento. Partiendo de la premisa de que las acciones que inciden únicamente en el ámbito biopsíquico individual son insuficientes para hacer frente al desgaste de la vida académica, se implementó la asignatura optativa “Afrontamiento del desgaste de la vida universitaria”, abierta a todos los estudiantes de grado de la Universidad de São Paulo, para proporcionar un espacio de discusión y escucha sobre las experiencias académicas, con miras a la construcción conjunta de estrategias para afrontar el desgaste y sus implicaciones en la formación universitaria. El presente estudio se propuso identificar los cambios en las concepciones de los estudiantes sobre el desgaste de la vida académica y sus causas, y elaborar junto con los estudiantes la propuesta de prácticas y estrategias para fortalecer la experiencia académica a lo largo de la asignatura.

## MÉTODO

### Tipo de Estudio

Se trata de un estudio descriptivo con un enfoque cualitativo basado en la investigación participante, producto de una tesis de maestría. En este enfoque, la relación objeto-investigador se sustituye por la relación sujeto-sujeto y, a través del diálogo, se comparten diferentes conocimientos que producen la comprensión de la realidad social y su transformación^(9)^.

### Sobre la Asignatura y los Sujetos de la Investigación

La asignatura se creó con el fin de superar las propuestas de acciones orientadas al fomento del autocuidado individual, en respuesta al desgaste, y se fijó los siguientes objetivos: a) Caracterizar a los diferentes jóvenes con distintos perfiles de reproducción social en la contemporaneidad; b) Identificar y analizar los potenciales de fortalecimiento/desgaste de la experiencia universitaria; c) Elaborar propuestas conjuntas para hacer frente a los potenciales de desgaste de la experiencia universitaria.

La disciplina se ofreció por primera vez en noviembre de 2023, vinculada a la Rectoría de la universidad, en modalidad optativa para los estudiantes y titulada “Afrontamiento del desgaste de la experiencia universitaria”. Se impartió en una de las 42 unidades de enseñanza de la Universidad de São Paulo, que cuenta con unos 60 000 estudiantes. Aunque solo se ofrecieron 30 plazas para estudiantes de todas las carreras de esta universidad, se inscribieron 776 estudiantes. Se optó por aceptar la selección del sistema de matriculación, que eligió a los primeros treinta estudiantes que dependían de los créditos de la asignatura para graduarse. Finalmente, participaron en la asignatura y en la investigación nueve estudiantes, ya que los demás habían interpretado que la oferta sería en formato remoto, al igual que la mayoría de las asignaturas vinculadas a la Vicerrectoría de Graduación^1^, y no pudieron participar de forma presencial.

Los sujetos de la investigación fueron los estudiantes que se inscribieron en esta asignatura y que aceptaron participar en la investigación. La invitación se hizo al final de la asignatura, después de la entrega de las calificaciones, y los estudiantes optaron por permitir que sus registros se utilizaran como material de análisis de la investigación, tras dar su consentimiento oficializado con la firma del Término de Consentimiento Libre e Informado (TCLE).

### Desarrollo de la Asignatura

La asignatura fue desarrollada por tres profesores de dos unidades de enseñanza distintas y un monitor. Se utilizaron estrategias y métodos activos para abordar los temas: juventud y contemporaneidad, la Universidad como organización social, la experiencia universitaria, la educación emancipadora como instrumento de mejora de la experiencia universitaria, estrategias para hacer frente al desgaste en la universidad, la participación política y los movimientos sociales como herramientas para hacer frente al desgaste.

Con el fin de identificar mejoras en las concepciones de los estudiantes sobre el desgaste de la vida académica y sus causas, se pidió a los estudiantes que respondieran, al inicio y al final del primer encuentro, a preguntas abiertas, siguiendo el modelo de pre y post test, a ejemplo del estudio de Cordeiro et al.^(10)^. Tras explicar el objetivo de esta actividad, los estudiantes dispusieron de unos 40 minutos para responder a las preguntas. En la primera sesión, las preguntas fueron: – En suexperiencia universitaria, ¿qué aspectos positivos de la vida académica identifica? – ¿Cuáles serían los desgastes y sus causas? – ¿Cuáles serían las formas de afrontar estos desgastes? En la última sesión de la asignatura se repitieron las preguntas de la primera sesión, añadiendo la siguiente: – ¿Crees que participar en la asignatura ha contribuido a tu proceso de formación? ¿En qué aspectos?

Las respuestas a estas preguntas, referidas al inicio y al final de la asignatura, se analizaron para identificar la contribución de la asignatura a la mejora de los conceptos y perspectivas de los estudiantes de licenciatura sobre los potenciales de desgaste/fortalecimiento de la experiencia universitaria y a la elaboración de propuestas para afrontarlos.

### Análisis de los Datos

Las respuestas de los estudiantes se analizaron mediante el método de análisis de contenido^(11)^. El análisis contempló la fragmentación de los registros en un proceso de construcción de unidades de significado o temas, a partir de los cuales se identificaron las categorías empíricas, con el fin de organizar e interpretar el contenido aportado en las respuestas de los estudiantes.

Tras la organización del contenido, el análisis se realizó utilizando los conceptos del campo de la Salud Colectiva.

### Aspectos Éticos

El proyecto matriz fue sometido al Comité de Ética e Investigación de la Escuela de Enfermería de la USP, que emitió un dictamen final favorable, con el número 6.749.093. La investigación cumplió con todas las especificaciones de la Resolución 466, de 12 de diciembre de 2012, que aprueba las directrices y normas reguladoras de las investigaciones con seres humanos. Se invitó a los participantes a leer y firmar el Término de Consentimiento Libre y Esclarecido (TCLE) que fue presentado por el proponente del proyecto principal. Todos aceptaron participar en la investigación.

## RESULTADOS

Todos los participantes tenían entre 22 y 23 años de edad, tres eran mujeres y seis eran hombres, y cursaban diferentes carreras/escuelas: Ciencias de la Computación, Audiovisual, Farmacia, Geología, Ingeniería Eléctrica y Enfermería. De ellos, seis estaban en el último semestre de la carrera y dos en el cuarto semestre, es decir, ya habían cursado la mitad de la licenciatura.

A continuación se presentan resúmenes del contenido de los registros individuales de los estudiantes de licenciatura realizados el primer día de clase, denominado pretest, y en la última sesión, denominado postest.

El Cuadro 1 presenta los temas obtenidos a partir de las respuestas a la pregunta sobre qué desgastes permean la vida académica y cuáles son las causas de esos desgastes.

**Cuadro 1:** Temas identificados en el pre-test y el post-test para la pregunta “¿Cuáles serían los desgastes y sus causas?” – São Paulo, SP, Brasil, 2024.

Temas PRE-TEST	Temas POST-TEST
Enfermedad psicológica de la comunidad académica Entorno académico competitivo y con altas expectativas Sobrecarga y sentimiento de culpa Elaboración de un plan de estudios con exceso de contenidos y sin áreas libres Dificultad para establecer relaciones interpersonales Falta de didáctica de los profesores y relaciones conflictivas entre docentes y estudiantes Insuficiencia de las políticas de permanencia para atender al perfil social de los estudiantes Sensación de pérdida de tiempo durante el desplazamiento a la universidad, que podría utilizarse para estudiar Sentimientos de inseguridad e incertidumbre Sentimiento de identificación y pertenencia perjudicado por los prejuicios Plantilla docente insuficiente	Enfermedad psicológica de la comunidad académica Estímulo del ambiente académico a la competitividad y el aislamiento Fomento del ambiente académico a la sobrecarga de actividades Falta de didáctica de los profesores y relaciones conflictivas entre docentes y alumnos Insuficiencia de las políticas de permanencia para atender al perfil social de los estudiantes Sensación de no pertenencia Valores neoliberales y exigencia de productividad académica

Es importante destacar aquí que dos temas llamaron la atención de los investigadores, ya sea porque estuvieron mucho más presentes al final de la asignatura (postest) en comparación con los registros del primer día de clase (pre-test), o porque se presentaron exclusivamente el último día, lo que denota cambios en las concepciones de los estudiantes sobre el potencial de desgaste y fortalecimiento de la vida académica, así como propuestas para hacer frente al desgaste identificado.

Así, la “**Insuficiencia de las políticas de permanencia para atender al perfil social de los estudiantes**” se identificó como una de las causas del desgaste en ambos momentos (pre y post test), pero en el último día de clase este tema fue planteado con mayor intensidad por los estudiantes. El tema “**Valores neoliberales y la exigencia del productivismo académico**”, por su parte, solo se mencionó en el post-test y los registros de los estudiantes explicitaron la reproducción de los valores neoliberales en la universidad, como la competitividad y el aislamiento, tal y como se puede identificar en el siguiente fragmento:

“Las principales razones de este desgaste están ligadas al estado de la sociedad capitalista moderna. El ambiente universitario se refleja en el ambiente externo, por lo que es perceptible su cambio durante los últimos años. El ambiente universitario es cada vez más productivista y competitivo, los profesores se ven obligados a publicar el mayor número posible de artículos para que la universidad pueda alcanzar las metas de productividad y obtener inversión del gobierno, priorizando la cantidad sobre la calidad (...)”

El Cuadro 2 contiene los temas obtenidos a partir de los registros de la pregunta sobre los aspectos positivos de la vida académica señalados por los estudiantes.

**Cuadro 2:** Temas identificados en en el pre-test y el post-test a la pregunta “En su experiencia universitaria, ¿qué aspectos positivos de la vida académica identifica?” – São Paulo, SP, Brasil, 2024.

Temas PRE-TEST	Temas POST-TEST
Redes de apoyo formadas por compañeros y familiares Buena relación con profesores y mentores Factores institucionales que contribuyen a la permanencia de los estudiantes Aprovechamiento de las oportunidades que ofrece la licenciatura Organización del plan de estudios que tiene en cuenta las necesidades de los estudiantes	Redes de apoyo formadas por compañeros y familiares (además de comentarios sobre el hecho de que la proximidad es mayor entre personas con las mismas condiciones sociales) Buena relación con profesores y mentores Factores institucionales que contribuyen a la permanencia de los estudiantes Aprovechamiento de las oportunidades que ofrece la licenciatura Organización del plan de estudios que tiene en cuenta las necesidades de los estudiantes Comprensión de la fuerza colectiva Comprensión de las cuestiones estructurales (políticas y económicas) que afectan a la universidad Comprensión de las causas del desgaste

Se observa que los tres últimos temas solo se encontraron en la prueba posterior, en la que las perspectivas de fortalecimiento individual se sumaron a las experiencias colectivas. Uno de los temas añadidos fue la **“Comprensión de la fuerza colectiva”**, en el que los estudiantes señalan que la participación política y la movilización colectiva promueven una lucha más eficaz contra el desgaste presente en el entorno universitario.

Otro tema señalado exclusivamente en la prueba posterior, **“Comprensión de las cuestiones estructurales (políticas y económicas) que atraviesan la universidad”,** sigue la lógica de que la comprensión de la universidad como una institución promotora de desgastes y fortalecimientos, reproductora de los valores en los que se basa la sociedad a la que pertenece, puede configurarse como un punto de fortalecimiento para los universitarios, tal y como explicó uno de los estudiantes el último día de clase:

“[La asignatura] me hizo comprender y darme cuenta de que cierta “culpa” que sentía por no haber hecho tantas cosas, en comparación con algunos compañeros, tiene una raíz y un origen que no proviene de mí. Además, la asignatura en su conjunto me hizo más consciente de los problemas de la vida académica actual. Aunque siempre están presentes, incluso en mi rutina, detenerme a pensar y aclarar cuáles son los desgastes que todos enfrentamos fue bueno para adquirir más autoconocimiento”.

El último tema, “**Comprensión de las causas del sufrimiento**”, se refiere a la comprensión de las raíces del desgaste de la vida universitaria, de modo que la culpa deja de ser exclusivamente del individuo al reconocer la influencia de los valores neoliberales que reproduce la universidad.

A continuación se presentan los temas relacionados con las propuestas de acciones y estrategias para hacer frente al desgaste identificadas por los participantes al inicio y al final de la asignatura (Cuadro 3).

**Cuadro 3:** Temas identificados en en el pre-test y el post-test a la pregunta “¿Cuáles serían las formas de hacer frente a este desgaste?” – São Paulo, SP, Brasil, 2024.

Temas PRE-TEST	Temas POST-TEST
Promoción de espacios de convivencia Participación en actividades extracurriculares Reorientación del plan de estudios Estrategias para atender las necesidades pedagógicas de los estudiantes Acceso a servicios de apoyo psicológico Políticas de permanencia más asertivas Estrategias asertivas para combatir los prejuicios y el acoso Reestructuración y formación pedagógica del cuerpo docente Estrategias individuales como afrontamiento (organización del tiempo entre los estudios y la convivencia familiar)	Promoción de espacios de convivencia y articulación entre estudiantes y personal administrativo, y entre docentes y personal técnico-administrativo Mejora de la formación y participación en actividades de extensión Reorientación del plan de estudios Estrategias para atender las necesidades pedagógicas de los estudiantes Acceso a servicios de apoyo psicológico Compromiso político y social en la universidad Disciplinas que promuevan espacios de reflexión sobre la universidad Uso de métricas cualitativas para evaluar la enseñanza en la universidad

Al final de la asignatura quedó claro hasta qué punto los estudiantes habían empezado a considerar las cuestiones colectivas y las respuestas políticas en el proceso de lucha contra el desgaste. En esta perspectiva, el tema “**Compromiso político y social en la universidad**” fue el más citado como forma de afrontar el desgaste, con seis estudiantes manifestándose sobre cómo la participación activa en movimientos políticos y sociales, en colectivos estudiantiles, en acciones que promueven la unión de los sectores que componen la comunidad universitaria, en círculos de conversación, en la representación estudiantil en los departamentos, son de suma importancia para la construcción de estrategias de enfrentamiento.

“La forma de enfrentar el desgaste es la unión de los tres sectores que componen la Universidad y la organización para cambios políticos, así como la realización de debates, reuniones y disciplinas que tengan como objetivo transferir cuáles son los desgastes y cuáles son sus causas, para que haya conciencia y contribuciones de todos”.

Cabe destacar la afirmación sobre la necesidad de participación de los tres sectores (estudiantes, docentes y personal técnico-administrativo) en los espacios de discusión. La representación estudiantil en comisiones y entidades colectivas estudiantiles de la universidad también se mencionó como estrategia de compromiso político.

Por último, el último tema/estrategia identificado solo en el análisis de la prueba posterior fue la oferta de **“Disciplinas que promuevan un espacio de reflexión sobre la universidad”**, en el que los estudiantes señalaron que la creación institucional de estos espacios promueve un potencial para hacer frente al desgaste.

## DISCUSIÓN

Entre los posibles desgastes enumerados por los estudiantes, registrados en la prueba previa, se relacionaron las condiciones materiales de existencia y permanencia de los estudiantes que interfieren directamente en la vida universitaria^(12)^.

Actualmente existen iniciativas que amplían los programas que favorecen la permanencia de los estudiantes. Sin embargo, incluso con la existencia de estos programas, como comedores universitarios y becas de alimentación, reconocidos por los estudiantes como potenciales fortalecedores de la vida académica, no son suficientes para responder de manera eficaz a la rápida transformación de los perfiles de reproducción social de los estudiantes que ingresan en las instituciones de educación superior(4,12,13).

Además de la necesidad de recursos que apoyen las políticas de permanencia en la universidad, también es necesario fortalecer las redes de apoyo y las relaciones interpersonales. La falta de apoyo familiar y la dificultad para establecer relaciones interpersonales son elementos que perjudican la experiencia universitaria^(9)^. La pertenencia a grupos, el apoyo familiar y las amistades contribuyen a afrontar las dificultades, ya que se sienten parte de colectivos^(4,12,14)^.

Desde la perspectiva de las relaciones interpersonales, es importante destacar que la competencia estimulada en el entorno académico debilita los lazos y las redes de apoyo, sustituyendo las relaciones solidarias por relaciones competitivas.

La “cultura de la competitividad” es uno de los aspectos negativos de la experiencia de los estudiantes universitarios^(12)^. La intensa competitividad observada en el ámbito académico se considera una consecuencia de la reproducción de los ideales neoliberales en la universidad^(13,15)^.

La sociedad neoliberal, en general, atribuye cada vez más al individuo toda la responsabilidad de su éxito o fracaso en todos los ámbitos de su vida. Esta lógica, reforzada por el proceso de individualización^(16)^, conlleva la ruptura de los vínculos colectivos, ya que las relaciones sociales se vuelven más vacías y con menos sentido. De este modo, recae exclusivamente en el sujeto la obligación de dedicarse lo suficiente para alcanzar sus objetivos^(15)^.

La dificultad de lidiar con esta responsabilidad excesiva, con la escasez de oportunidades y con el abandono por parte de las instituciones conduce a un sentimiento de impotencia y culpa, lo que potencia el sufrimiento por no alcanzar el nivel impuesto como ideal^(7,13,15)^.

En la universidad, este contexto se ve agravado por el “productivismo académico”, fenómeno que se estructura en la lógica de la valoración excesiva de la cantidad de producción científica y académica en detrimento de su calidad^(17,18)^.

La universidad está inmersa en el conjunto de relaciones sociales del modo de producción hegemónico; por lo tanto, se rige por la sociabilidad capitalista, en la que son constitutivos elementos como la mercantilización y la burocratización de las relaciones sociales. La Universidad deja de orientar sus prácticas de formación de trabajadores e investigación para responder a las necesidades de la sociedad, para dar prioridad a las necesidades del mercado, la producción de mercancías. De esta manera, la universidad pública asume su carácter de universidad operativa, que prioriza la individualización, el rendimiento y la eficacia^(19)^.

Los estudiantes que participaron en este estudio identificaron como uno de los factores de desgaste de la vida académica la falta de didáctica de los profesores, lo que repercute directamente en la calidad de la enseñanza y en la disponibilidad del profesor para ser docente, además de ser un gran generador de desgaste para los estudiantes de licenciatura. La dificultad para realizar la transición del contenido teórico al práctico, las estrategias pedagógicas obsoletas, la falta de atención a los estudiantes y la falta de actualización de los conocimientos provocan descontento con respecto al curso^(20)^.

Un agravante es que también hay falta de diálogo y de escucha por parte de los profesores y los estudiantes sobre los procesos de construcción de la matriz curricular^(20)^. La intensa carga horaria o el período integral, la acumulación de actividades obligatorias y la carga horaria de las universidades son causas de desgaste^(4)^.

Así, se verifica que el desgaste en la vida académica se relaciona con cuestiones que van más allá de las dificultades individuales de los estudiantes y que tienen que ver con el proceso de adaptación a la vida académica. Se refieren a elementos estructurales que implican, entre otros aspectos, la falta de atención a las distintas necesidades de los estudiantes determinadas por sus perfiles de reproducción social, la relación jerárquica entre profesores y alumnos y la organización de la matriz curricular.

Por otro lado, las universidades, al identificar las manifestaciones del desgaste en el sufrimiento psíquico, ofrecen estrategias de atención, predominantemente curativas, es decir, centradas en los síntomas, y acciones centradas en el autocuidado^(1,14)^. El fenómeno se homogeneiza e individualiza^(6,17)^.

Es necesario pensar en estrategias colectivas que permitan afrontar institucionalmente el sufrimiento psíquico de estos jóvenes universitarios^(1)^, tal y como señalan los estudiantes. El compromiso político y social y la organización de los sujetos en grupos, en torno a un objetivo/tema común, representan una forma de combatir el proceso de individualización. Para que estos espacios permitan el desarrollo de instrumentos para hacer frente al desgaste, deben tenerse en cuenta las dimensiones colectivas de los orígenes del sufrimiento^(7)^, tal y como se registró en la prueba posterior.

Es importante que se implementen acciones colectivas en el entorno académico, ya que tienen un potencial de fortalecimiento y transformación. Los espacios colectivos promueven la salud y cuestionan las conductas que enferman a los estudiantes^(21)^. Los encuentros proporcionan el poder de actuar y, en consecuencia, de hacer frente al desgaste.

## CONCLUSIÓN

A partir de los registros de los estudiantes, se verificó que la disciplina provocó reflexiones sobre cómo la participación política y social, además de la organización de los sujetos en grupos, es un importante potencial de fortalecimiento para la experiencia académica, impregnada por la lógica desgastante y enfermiza de la sociabilidad capitalista.

La asignatura contribuyó a que los estudiantes perfeccionaran sus concepciones sobre el desgaste de la experiencia universitaria y sus causas, sobre los potenciales de fortalecimiento durante la licenciatura como formas de proponer un enfrentamiento a los potenciales de desgaste.

Desde esta perspectiva, se identificaron mejoras en lo que respecta a la perspectiva colectiva de la confrontación, explicando la necesidad de actuar sobre las raíces del desgaste, a través de una participación política activa.

El presente trabajo tuvo como limitación el número de participantes. Es importante que se realicen nuevos estudios a partir de futuras ofertas de la disciplina con otros criterios de selección para ampliar el número de estudiantes.

Se postula que la disciplina debe ofrecerse de manera continua, propiciando un espacio de reflexión, discusión y facilitación de acciones colectivas de fortalecimiento de la vida académica.

## Data Availability

El conjunto de datos que respaldan las conclusiones de este estudio está disponible previa solicitud al autor correspondiente.
